# Evidence of hydrogen trapping at second phase particles in zirconium alloys

**DOI:** 10.1038/s41598-021-83859-w

**Published:** 2021-02-23

**Authors:** Christopher Jones, Vidur Tuli, Zaheen Shah, Mhairi Gass, Patrick A. Burr, Michael Preuss, Katie L. Moore

**Affiliations:** 1 Department of Materials, University of Manchester, Manchester, M13 9PL UK; 2 School Mechanical and Manufacturing Engineering, University of New South Wales, Sydney, NSW 2052 Australia; 3 Westinghouse Electric Sweden AB, 721 63 Västerås, Sweden; 4 Jacobs, Walton House, Warrington, WA3 6GA UK; 5 Monash University, Clayton, VIC 3800 Australia; 6 Photon Science Institute, University of Manchester, Manchester, M13 9PL UK

**Keywords:** Metals and alloys, Electronic structure, Mass spectrometry

## Abstract

Zirconium alloys are used in safety–critical roles in the nuclear industry and their degradation due to ingress of hydrogen in service is a concern. In this work experimental evidence, supported by density functional theory modelling, shows that the α-Zr matrix surrounding second phase particles acts as a trapping site for hydrogen, which has not been previously reported in zirconium. This is unaccounted for in current models of hydrogen behaviour in Zr alloys and as such could impact development of these models. Zircaloy-2 and Zircaloy-4 samples were corroded at 350 °C in simulated pressurised water reactor coolant before being isotopically spiked with ^2^H_2_O in a second autoclave step. The distribution of ^2^H, Fe and Cr was characterised using nanoscale secondary ion mass spectrometry (NanoSIMS) and high-resolution energy dispersive X-ray spectroscopy. ^2^H^−^ was found to be concentrated around second phase particles in the α-Zr lattice with peak hydrogen isotope ratios of ^2^H/^1^H = 0.018–0.082. DFT modelling confirms that the hydrogen thermodynamically favours sitting in the surrounding zirconium matrix rather than within the second phase particles. Knowledge of this trapping mechanism will inform the development of current understanding of zirconium alloy degradation through-life.

## Introduction

The degradation of materials as a result of hydrogen ingress is a point of concern for safety critical components across many industries, such as nuclear^1–4^, aerospace^5,6^, and oil & gas^7^. Hydrogen has been shown to cause cracking^4,5,8^, hydride precipitation^1–3^, embrittlement^9^, and other such deleterious effects^10^, limiting the useful life of components in service and increasing both operating cost and the probability of unsafe conditions arising during use. This is especially critical in nuclear power systems that see heavy use of zirconium alloys as both fuel cladding and structural materials^11^. Zirconium alloys typically form a crucial part of the safety systems inside a reactor core and these alloys are prone to high levels of hydrogen pick-up as a result of corrosion during service^12^.

Zircaloy-2 (Zy-2) [1.2–1.7 wt% Sn, 0.07–0.2 wt% Fe, 0.05–0.15 wt% Cr and 0.03–0.08 wt% Ni, balance Zr] and Zircaloy-4 (Zy-4) [1.2–1.7 wt% Sn, 0.18–0.24 wt% Fe and 0.1–0.13 wt% Cr, balance Zr] are zirconium alloys commonly used as the fuel cladding in pressurized and boiling water nuclear reactors due to their relative transparency to neutrons and their good mechanical and corrosion properties. In these environments the alloys are exposed to high temperatures (300–350 °C), high radiation fields (primarily neutron radiation) and an aqueous environment. As a result of these combined environmental factors Zy-2 and Zy-4 undergo degradation in service through oxidation and hydrogen ingress.

Zirconium forms a protective oxide during corrosion, and once an initial oxide film is formed further oxidation takes place via the transport of oxygen to the metal/oxide interface^13–15^. Oxygen is typically provided through the reduction of water molecules to produce O^2−^ ions, a process that also produces H^+^ ions. These H^+^ ions adsorb onto the surface of the oxide layer and then either combine with electrons, released during the oxidation of Zr atoms, to form H_2_ molecules that escape the system as gas, or diffuse through to the Zr metal substrate^16–18^. Current literature is unclear on the reasons for hydrogen ingress into the metal substrate, with some recent models claiming the H^+^ ions play a role in balancing the charge during the oxidation reaction^19^. The presence of hydrogen in the metal causes degradation of the fuel cladding, typically either by hydrogen embrittlement or delayed hydride cracking^2,3,20,21^.

Second phase particles (SPPs) are formed from Fe, Cr and Ni alloying elements present in Zy-2 and from Fe and Cr alloying elements in Zy-4 as these elements have very low solubility in the α-Zr lattice. The distribution and size of SPPs is known to impact the corrosion rate and subsequent introduction of hydrogen into the base metal, however the exact mechanisms and effects of SPPs on corrosion and hydrogen pick up are still under debate^19,22–26^.

Previous work looking at the distribution of H within α-Zr has typically used techniques such as SIMS^21,22,27^ and nuclear analysis techniques^19,28–32^. Both of these techniques tend to have limited spatial resolution, which prevents the observation of small features such as SPPs and any associated hydrogen concentrations. The NanoSIMS provides the combination of high resolution and high sensitivity necessary for the detection of ^2^H associated with SPPs, something which earlier SIMS techniques could not achieve. Previous NanoSIMS analysis of hydrogen distributions in zirconium alloys is limited to date. Yardley et al.^33^ investigated ^2^H doped ZIRLO but did not find deuterium associated with SPPs, however a Nb containing ZIRLO alloy does not have SPPs with the same composition as seen in Zy-2 and Zy-4. Li et al.^34^ performed 3D characterisation of deuterium distributions in Zy-4 but this analysis was primarily limited to the Zr oxide^34^.

Atom probe tomography (APT) has the potential to detect hydrogen atoms in a sample, and several studies^35–39^ have investigated SPPs in various zirconium alloys, both in the zirconium metal and the oxide layer. None of these studies have reported hydrogen interacting with SPPs, either in the bulk of the SPP or the precipitate. However, this may be due to the manner in which atom probe samples are prepared, commonly using focussed ion beam milling, which damages trapping sites and allowed hydrogen to escape. This would be exacerbated by the high surface area to volume ratios of atom probe samples allowing for hydrogen to easily escape when freed from trap sites, unless prepared, stored and transported under appropriate cryogenic conditions^40^. Recent modelling work has used density functional theory (DFT) to simulate the thermodynamics of hydrogen segregation to binary SPPs in an α-Zr lattice, i.e. ZrFe_2_ or ZrCr_2_ SPPs^25,41^. This work found that the most favoured site for hydrogen storage was in tetrahedral sites with a high Zr/M ratio (where M was any of the other metallic elements) with SPP structure having less of an impact, agreeing with the previous models^42^.

However it also concluded that the enthalpy of hydrogen accommodation in Zr(Fe,Cr)_2_ SPPs was unfavourable compared to accommodation of hydrogen in the α-Zr lattice^25,41^. It is important to note though that this modelling of SPPs as binary ordered structures is not representative of the reality that Zr(Fe,Cr)_2_ SPPs are ternary, partially disordered structures. The disorder in the structure is expected to provide many different local environments, which would then in turn provide a subtly different H solution energy. Precipitates have been widely studied in other materials for their interactions with hydrogen. Many commonly used Al, Fe and Ni alloys have been reported to show hydrogen trapping either at interfaces between precipitates and the matrix, or within the precipitates^43–47^, with varying mechanisms and depth of the trap sites. Despite the importance of hydrogen-based degradation on the performance of zirconium alloys in nuclear contexts, there are no current papers investigating SPP interaction with hydrogen in a zirconium alloy matrix.

In this work the distribution of deuterium in two zirconium alloys was investigated using high resolution NanoSIMS. Complementary DFT modelling was used to investigate whether a range of different local environments would provide favourable sites for H in the SPPs when compared to the Zr lattice. The approach adopted here, combining high resolution NanoSIMS (able to directly detect and map hydrogen) with targeted computer modelling, presents a potential model for analysis of hydrogen behaviour in other alloys and safety critical components.

## Results

### Experimental

**Figure 1:**
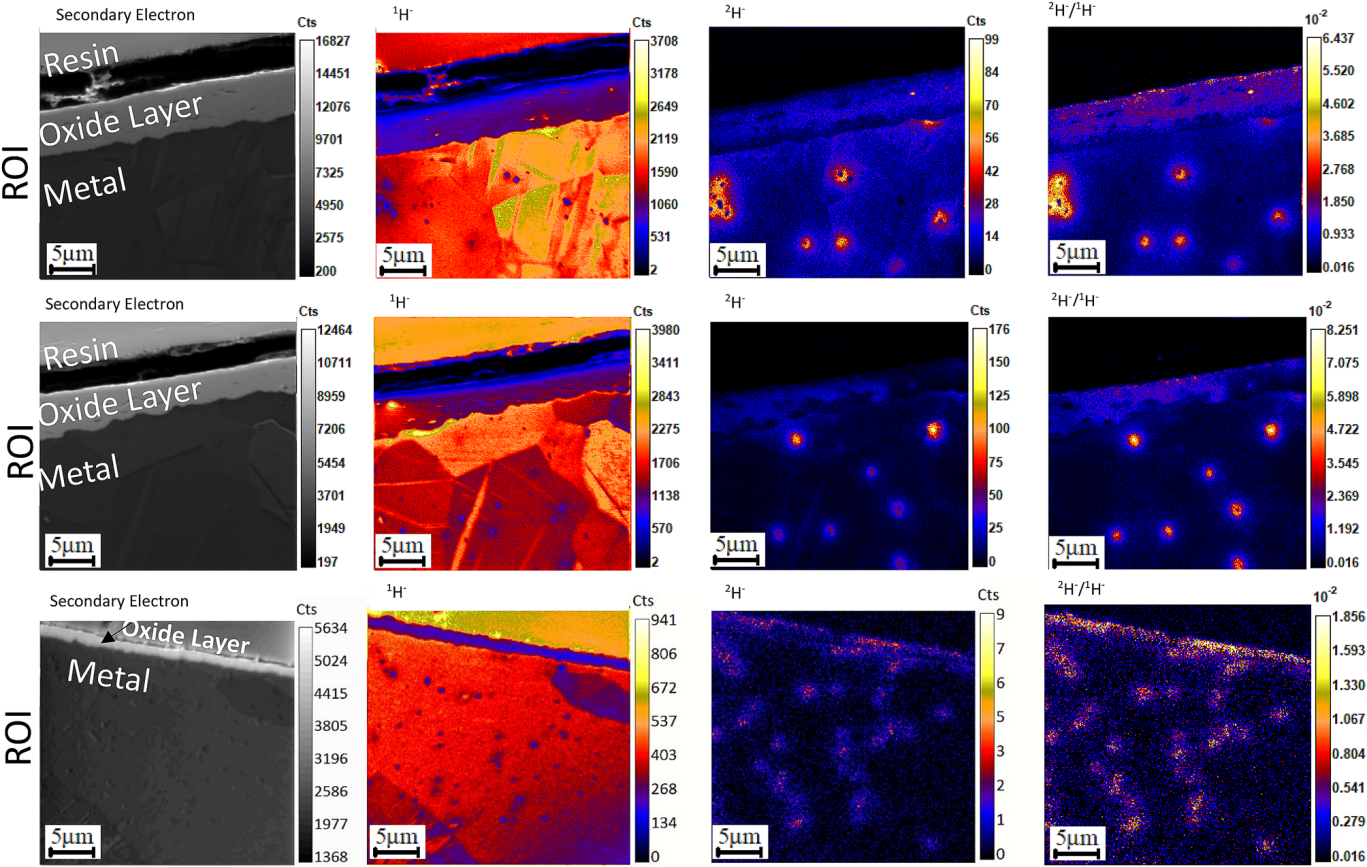
Secondary electron, ^1^H^−^, ^2^H^−^ images and the ^2^H^−^/^1^H^−^ ratio for three separate regions of interest (ROIs) showing the total counts summed over 10 image planes for ROIs 1 & 2 and 5 image planes for ROI 3. ROIs 1 and 2 are from the Zy-4 sample and ROI 3 is from the 48.3 day Zy-2 sample. ROIs 1 and 2 were implanted with 5.7 × 10^17^ ions/cm^2^ prior to imaging and imaged with currents of 1.79 pA and 1.75 pA respectively, a dwell time of 5000 μs/pixel and 256 × 256 pixels over a 30 μm raster size. ROI 3 was implanted with 3 × 10^17^ ions/cm^2^ prior to imaging and imaged with a current of 1.57 pA, a dwell time of 5000 μs/pixel and 256 × 256 pixels over a 30 μm raster size.

To investigate the distribution of hydrogen and deuterium in Zircaloy samples, several (> 10 in total) regions of interest (ROIs) were imaged by NanoSIMS. Figure 1 shows the Secondary Electron (SE), ^1^H^−^, ^2^H^−^ and ^2^H^−^/^1^H^−^ ratio images for three separate ROIs on the 402.2 day Zy-4 (ROIs 1,2) and 48.3 day Zy-2 samples (ROI 3). The ^1^H^−^ images reveal the grain structure of the sample; the signal variation in different grains is due to the variation in sputter rate with grain orientation^48^. Taking a ratio of the ^2^H^−^ and ^1^H^−^ signals effectively allows for differences in the ion signal due to differences in the grain to grain sputter rate to be cancelled out as the variation in sputter rates will be consistent between hydrogen isotopes. Also visible in the ^1^H^−^ image are several small, dark circular features with a lower signal compared to the surrounding metal. These dark features appear to be SPPs in the α-Zr matrix. The ^2^H^−^ images show an inhomogeneous ^2^H distribution with small regions with high ^2^H abundance in the α-Zr matrix beneath the oxide layer. The same distribution is also clear in the ^2^H^−^/^1^H^−^ ratio images where grain orientation contrast has been minimised by ratioing. Both the Zy-4 and Zy-2 samples show the same localisation of ^2^H^−^ to the SPPs, but lower ^2^H^−^/^1^H^−^ ratios were observed in the Zy-2 sample. The Zy-4 and Zy-2 were oxidised at different times in different autoclaves yet still show the same localisation of ^2^H^−^ to the SPPs.

Table 1 shows the average size of the features visible in the ^2^H^−^/^1^H^−^ ratio images in Fig. 1. The sizes of these features were determined using L’Image (L.R. Nittler, Carnegie Institution of Washington) to generate circular boundaries around local maxima in the ^2^H^−^/^1^H^−^ distribution, with the threshold value for determining the placement of a boundary set at 20% of the global maximum ^2^H^−^/^1^H^−^ ratio in the metal substrate region of the ROIs in Fig. 1 (i.e. discounting any signals from the oxide and metal/oxide interface regions and any peaks in ratio below 20% of the maximum ratio). The outer edge of the circular ROIs was determined by the point at which the signal dropped below 50% of the local peak value. Additionally for each ROI shown in Fig. 1 the NanoSIMS beam width was determined using the 16–84% criterion^49^.

**Table 1:** Table of ^2^H^*−*^/^1^H^*−*^ feature sizes and NanoSIMS beam widths for ROIs in Fig. 1.

Sample	^2^H^−^/^1^H^−^ feature size (μm)	Beam width (µm)
ROI 1	1.77 ± 0.42	0.26
ROI 2	1.45 ± 0.10	0.20
ROI 3	0.97 ± 0.27	0.21

**Figure 2:**
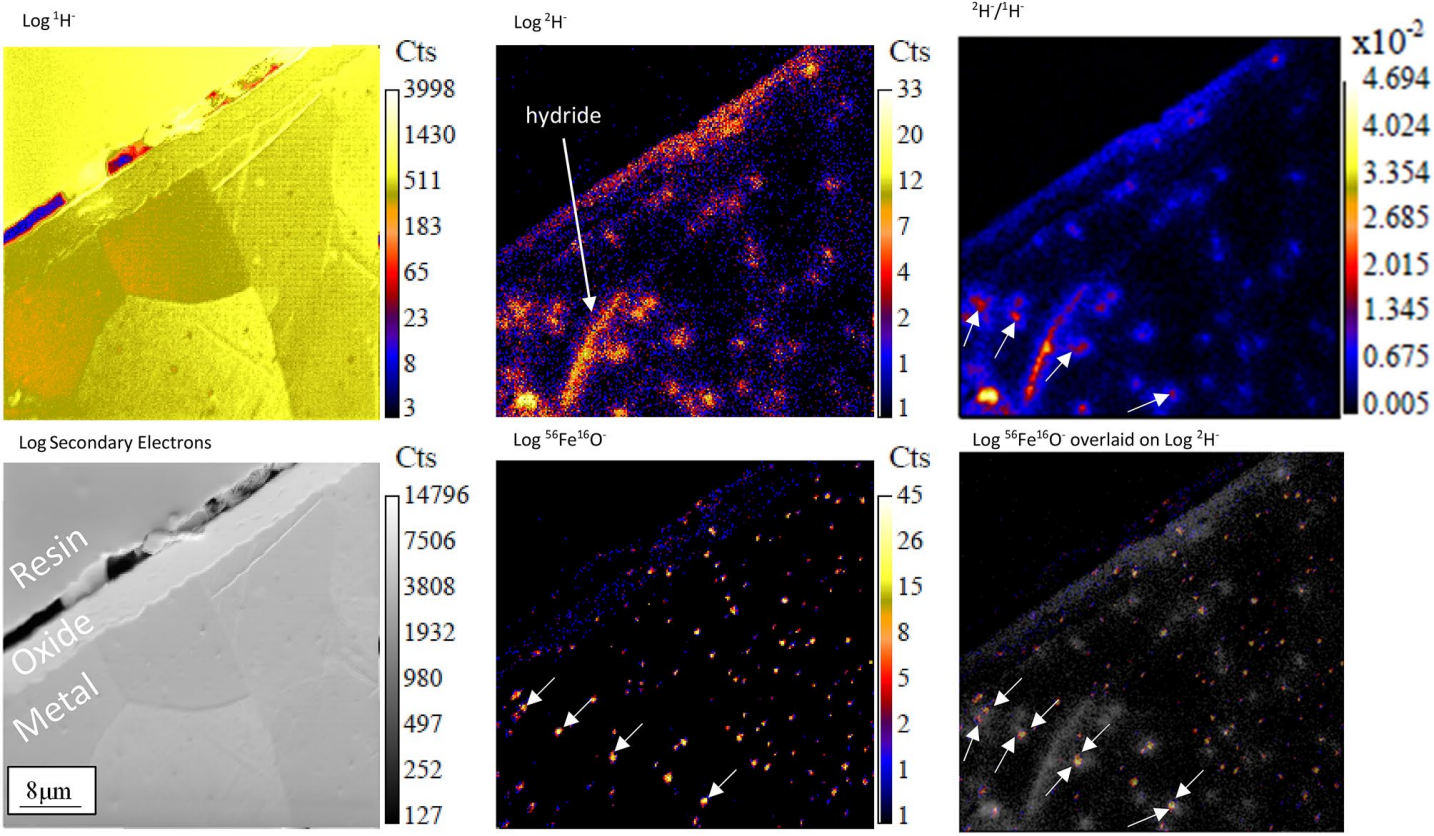
^1^H^−^, ^2^H^−^, Secondary electron and ^56^Fe^16^O^−^ images, the ^2^H^−^/^1^H^−^ ratio and an image of the ^56^Fe^16^O overlaid on ^2^H^−^ image of ROI 4 from the Zy-4 sample showing the total counts summed over 6 image planes. The ROI was implanted with 4.0 × 10^17^ ions/cm^2^ prior to imaging and imaged with a current of 1.34 pA, a dwell time of 4000 μs/pixel and 256 × 256 pixels over a 50 μm raster size.

Figure 2 shows the ^1^H^−^, ^2^H^−^, ^56^Fe^16^O^−^, SE and ^2^H^−^/^1^H^−^ ratio images from a fourth ROI on the Zy-4 sample, along with the ^56^Fe^16^O^−^ image overlaid on the ^2^H^−^ image. It can be observed in both the ^2^H^−^ image and the ^2^H^−^/^1^H^−^ ratio image (indicated with arrows in the latter) that there is again a pattern of localised ^2^H concentrations in the α-Zr region beneath the oxide, as well as the presence of a hydride in the lower left corner. Additionally, the distribution of ^56^Fe^16^O^−^ signal, representative of the Zr(Fe,Cr)_2_ SPPs, can be seen to match the distribution of the dark features in the ^1^H^−^ image, indicating that these dark features in the ^1^H^−^ image are SPPs. When the ^16^Fe^16^O^−^ image and the ^2^H^−^/^1^H^−^ ratio image are overlaid, the indicated ^56^Fe^16^O and ^2^H rich regions overlap.

**Figure 3:**
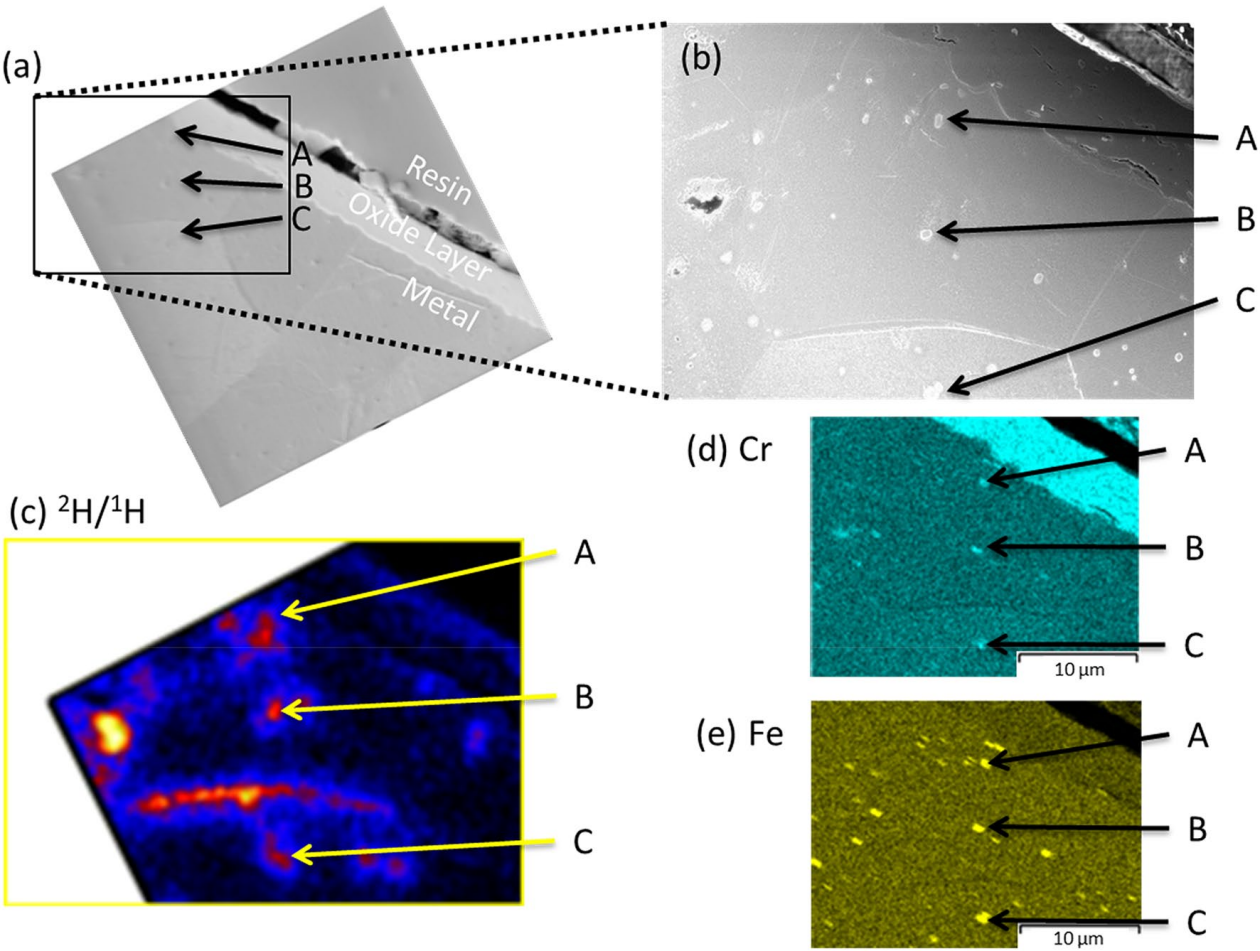
Correlative imaging between the NanoSIMS and EDX from the Zy-4 sample. (**a**) NanoSIMS secondary electron image shown in Fig. 2 rotated and marked to line up exactly with the secondary electron image from the FEG-SEM (**b**). Indicated on these images are three SPPs (A–C). The ^2^H/^1^H NanoSIMS ratio image (**c**) has been rotated and cropped to again line up with the EDS results obtained from the FEG-SEM. (**d**,**e**) show the Cr and Fe distributions respectively indicating the positions of SPPs.

Figure 3a,b shows SE images obtained using the NanoSIMS and an FEG-scanning electron microscope (SEM) respectively from the Zy-4 sample. The ^2^H^−^/^1^H^−^ ratio image for this is shown in Fig. 3c. This corresponds to the lower left corner of the ^2^H^−^/^1^H^−^ ratio image shown in Fig. 2 but the image has been rotated, cropped and resized to match with the SEM and Energy dispersive X-ray spectroscopy (EDS) images (Fig. 3b–d). Figure 3d,e show EDS Cr and Fe distribution maps of the same region with the SPPs labelled A–C matching the elevated ^2^H^−^/^1^H^−^ ratio distribution labelled A–C in Fig. 3c.

### Modelling

SPP crystal structures were generated with Fe: (Fe + Cr) atomic ratios of 43.75 at% and 62.50 at%. This is representative of the SPPs found in Zy-2 and Zy-4, respectively. No disorder was imposed onto the Zr sublattice.

**Figure 4:**
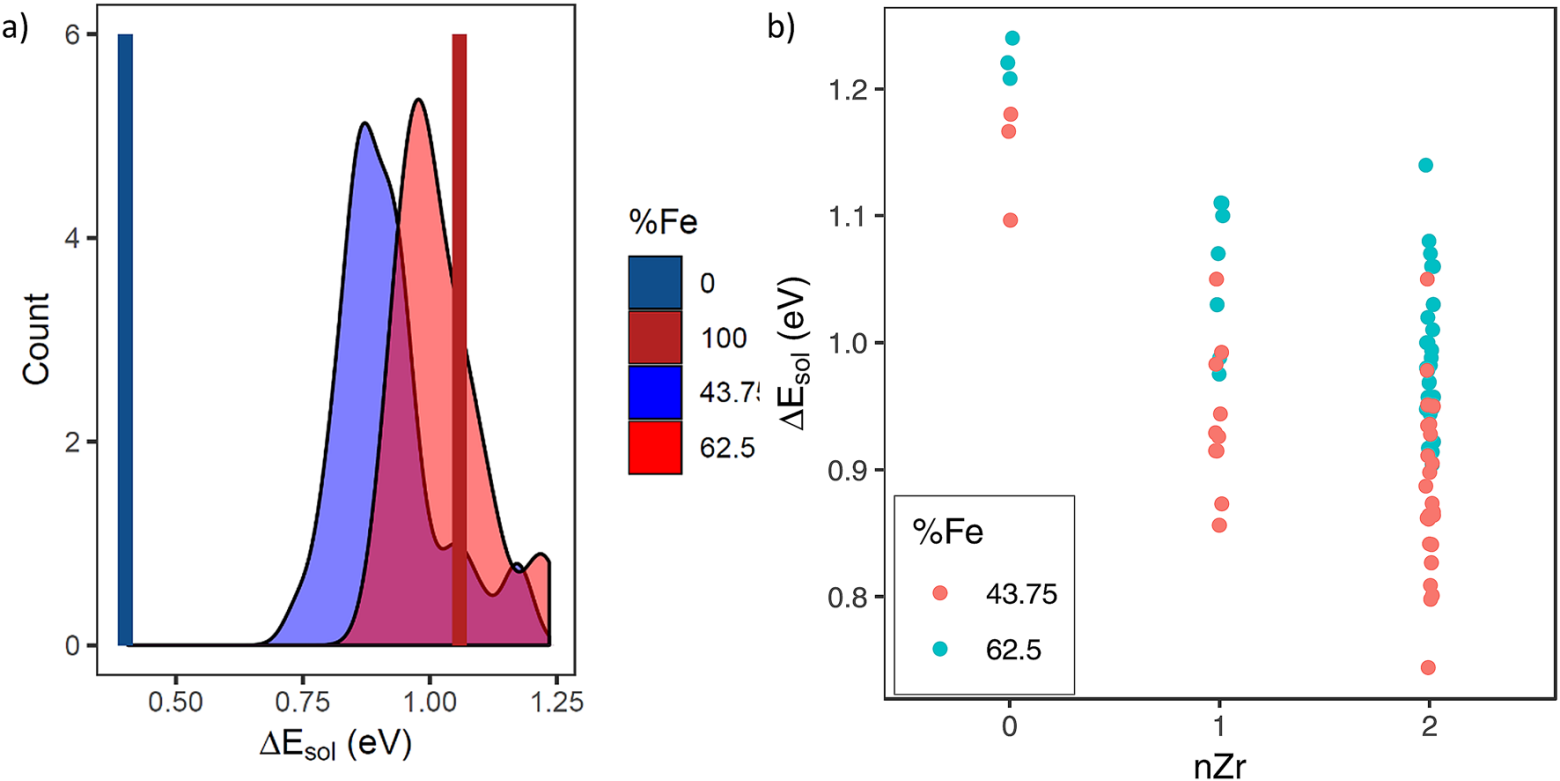
(**a**) Enthalpy difference for H solution in ternary SPPs (as depicted by the two broad peaks), ZrCr_2_ (blue line) and ZrFe_2_ (red line) when compared to H solution in hcp-Zr. (**b**) Relative enthalpy of solution of H in SPPs (compared to hcp-Zr) as a function of number of Zr nearest neighbour atoms (nZr).

Figure 4a shows the relative hydrogen solution enthalpy, ΔH_sol, for H to dissolve in the SPP compared to hcp-Zr, for 35 interstitial sites at each SPP composition. While the spread is significant, the relative solution energy is positive for all sites, as such these results still suggest that it is thermodynamically unfavourable for H to be in the bulk of the SPPs, compared to the bulk of hcp-Zr.

Analysis of the local chemistry (nearest neighbour configuration) revealed that for all simulations, H relaxed into a tetrahedral site, irrespective of starting coordinates. This matches previously observed behaviour in binary SPPs and α-Zr.

## Discussion

Figure 1 shows that the ^2^H is not evenly distributed throughout the α-Zr matrix and forms several ‘hot spots’ in all 3 ROIs. In ROIs 1 and 2, small dark features can be observed inside several of the areas with high ^2^H^−^ signal on the ^2^H image. The ^2^H^−^ signal strength in ROI 3, the 48.3 day Zy-2 sample, is too low to resolve these features to the same degree as for ROIs 1 and 2. It is proposed that the dark features visible in several of the ^2^H concentrations are SPPs, and that the ^2^H sits in the matrix around the SPPs.

The natural ^2^H/^1^H ratio is 1.56 × 10^–4^; however, ^2^H^−^/^1^H^−^ ratios reach values of 1.8–8.2 × 10^–2^ at the ^2^H^−^ enriched regions, which is more than 100 times higher than the natural background level. This high level of enrichment indicates that the deuterium detected originates from the oxidation process in the spiked autoclave rather than as part of the natural atmosphere present in the NanoSIMS chamber or sample preparation. The water used in the spiked autoclave was not 100% D_2_O so it was expected that elevated levels of ^1^H would also be observed around each of the SPPs. Due to high levels of ^1^H contamination of the cross sectioned surface from sample preparation and the vacuum chamber, elevated signals of ^1^H were not observed. The counts in the ^1^H image are more than an order of magnitude higher than the ^2^H counts, therefore any ^1^H enrichments that would be present after the autoclave experiment at a similar count rate to the ^2^H signal (for the 50% D2O labelled Zr-4) are masked by the background. As the H picked up during corrosion is observed to migrate to SPPs, no ^2^H enrichment is detected in regions between the SPPs. It is noted that as the samples were removed from the spiked autoclave for > 6 months before NanoSIMS analysis was conducted, deuterium present in the α-Zr matrix had sufficient time to redistribute towards more energetically favourable sites. The matrix around the SPPs can therefore be considered as a deep trap site as elevated ^2^H signals were still detectable.

Two separate approaches were taken to confirm the presence of SPPs at the locations of the high ^2^H/^1^H ratio. First the NanoSIMS was used to map the distribution of ^56^Fe^16^O^−^secondary ions in the sample. Fe^−^ has a low ionisation rate when using a Cs^+^ primary beam to investigate negative ions in the NanoSIMS, so ^56^Fe^16^O^−^ was used as a proxy signal for the Fe concentration. There is typically enough oxygen present in the NanoSIMS to allow for the continued oxidation of material newly uncovered by sputtering and as such the ^56^Fe^16^O^−^signal is proportional to the amount of Fe present.

By comparing the ^2^H^**−**^/^1^H^**−**^ ratio signal map in Fig. 2 with the ^56^Fe^16^O^−^ signal map, a correlation can be drawn between areas of high ^56^Fe^16^O^−^ signal, indicating SPPs, and the concentrations of high ^2^H^−^ signal. This is clearer in the overlay image, where the ^56^Fe^16^O^−^ signal can be seen to overlap with the majority of the ^2^H concentrations in the ROI. Not all areas of high ^56^Fe^16^O^−^ signal are co-located with high ^2^H^−^ signal, however there seems to be a strong correlation, with 68% of the ^2^H^−^ signal hotspots being associated with high ^56^Fe^16^O^−^ signal (Fig. 2). However, as discussed previously, the ^2^H enrichment is associated with the periphery of the SPPs rather than the SPPs themselves.

This fits with the DFT results presented here. Figure 4a shows that it is thermodynamically unfavourable for H to be accommodated inside Zy-2 or Zy-4 SPPs, whether the SPP is binary or ternary in composition, when in competition with the α-Zr matrix. It should also be noted that while these simulations have been performed without considering the effect of temperature, it is unlikely that the entropy contributions at the temperatures encountered in service (~ 300 °C) would be large enough to overcome the energy gap compared to hcp-Zr (0.70 eV). The predicted preference for hcp-Zr over SPPs is reinforced by further analysis of hydrogen’s local chemistry. Figure 4b shows that H preferentially occupies sites with the largest fraction of Zr nearest neighbours. It should be noted that in the structure of Zr(Fe,Cr)_2_ SPPs there are no tetrahedral sites bound by more than 2 Zr atoms. Even for these sites, favourable thermodynamics are not predicted. This information when combined with the distribution of ^2^H observed in the experimental results implies that the ^2^H is either trapped at the interface between the SPPs and the matrix or it is trapped in the matrix surrounding the SPPs.

Trapping of hydrogen at incoherent interfaces has been observed in other alloy systems, where incoherent interfaces between precipitates and the matrix have trapped hydrogen^40,43–47,50^. However, the DFT results shown here and presented in the literature^25,41^ indicate that the α-Zr matrix is the preferred site for hydrogen to inhabit.

The DFT analysis of hydrogen’s local chemistry indicates that the trapping of H around SPPs is probably not due to chemical affinity with Fe and Cr. Considering that H preferentially occupies tetrahedral sites in both the SPP and the α-Zr matrix, and since H shows a clear preference for tetrahedral sites with fewer Fe and Cr nearest neighbours, it is unlikely that the trapping in that region is due to direct bonding between H and Fe or Cr. On the other hand, it is well established that H is attracted to areas of tensile strain^51^.

Tensile stress fields may be present in the region surrounding the SPP-matrix interface due to a mismatch in thermal expansion coefficients between the SPPs and the matrix^52^. The coefficients of thermal expansion for ZrCr_2_^53^ and ZrFe_2_^54^ (both commonly observed stoichiometries of SPPs^25^) are both significantly larger than that for Zy-4^55^. As a result, when the alloy is cooled from processing or operating temperatures this results in the formation of tensile stresses in the matrix around the interface^52^. These tensile stresses could then attract hydrogen to the stressed matrix surrounding the SPPs as the system cools from elevated temperatures. The build-up of stresses as a result of thermal expansion coefficient mismatch has previously been observed for Al_2_Cu particles in an Al matrix^56^.

The enriched features in ROI 3 of Fig. 1 are noticeably smaller but greater in number, and have lower ^2^H^**−**^/^1^H^**−**^ ratios when compared to ROIs 1 and 2. This is also shown in Table 1 where the average feature size is smaller in ROI 3 despite the beam size and raster size being similar to ROIs 1 and 2. The ^1^H images in Fig. 1 each show a distribution of small dark features in the metal substrate, these are consistent with SPPs, with the SPP distribution in ROIs 1 and 2 mostly comprised of a small number of SPPs distributed in clusters whereas the SPPs in ROI 3 are present in greater numbers and are evenly distributed across the ROI. This matches the differences in the ^2^H^−^/^1^H^−^ features between the ROIs. The availability of a larger number of potential trapping sites (i.e. SPPs), a shorter exposure time to ^2^H_2_O (45 vs 60 days) and a lower spiking level (15 vs 50%) could explain why less ^2^H is concentrated in any given site in Zy-2, compared to Zy-4, and thus why the measured ^2^H^**−**^/^1^H^**−**^ ratio is lower. Additionally, the presence of Ni in Zy-2 compared with Zy-4 will change the stoichiometry of the SPPs, resulting in different coefficients of thermal expansion. This would lead to different strain fields around each SPP, potentially changing the ability of the matrix around the SPP to trap hydrogen.

In order to corroborate the NanoSIMS results, EDS analysis of the same ROI analysed in Fig. 2 was undertaken (Fig. 3). In the SE images a group of features < 1 µm in size are visible and scattered throughout the Zr grains (Fig. 3a,b), three of these features have been labelled A, B, and C. These features are surrounded by large amounts of ^2^H^−^ relative to the matrix away from them (Fig. 3c). EDS analysis shows that all these features are rich in Fe and Cr signals (Fig. 3d,e). The combination of their small size and the presence of Fe and Cr in the same location confirms that these features are SPPs with associated high ^2^H^−^ signals.

The phenomenon of hydrogen trapping in the matrix around SPPs has not previously been observed. While some of this can be attributed to an inability to detect hydrogen with commonly used SEM based techniques or a lack of spatial resolution in techniques commonly used to detect hydrogen, as detailed in the introduction to this paper, this does not fully explain why segregation of H to SPP interfaces has not been observed before.

Recent work by Li et al.^34^ and Liu et al.^57^ used NanoSIMS to investigate the distribution of ^2^H in zirconium oxides^34,57^ but did not observe the same trapping behaviour as seen here. This is due to the effect of the NanoSIMS analysis itself on the trapping sites, as beam damage and mixing from the 16 keV Cs^+^ ion beam destroys the trapping sites below the sampling depth of the NanoSIMS, allowing ^2^H to escape before being detected.

The beam damage renders it difficult to detect ^2^H around the SPPs as the trapping sites are irreversibly damaged during analysis, freeing the trapped ^2^H and makes the ^2^H trapping appear transient when analysed. The damage to trapping sites, and consequent spreading/escape of hydrogen, is reflected in the size of the features reported in Table 1. While the nature of this irradiation damage has not yet been analysed, it seems reasonable to expect the stress state in the matrix to change as a result of ion bombardment, potentially removing or reducing the tensile stresses that attracted hydrogen.

Table 1 gives a ^2^H concentration size of 0.97–1.77 μm, far larger than values for typical SPP size given in the literature^58^ where the modal size for SPPs in Zy-4 is 0.2 µm. This can be partly explained by the fact that the NanoSIMS beam size is much larger than the SPP but even when this is accounted for, the minimum size of the ^2^H features is measured at 0.28–0.95 μm, still larger than the expected size for SPPs in Zy-2 and Zy-4. This size increase is the result of ^2^H spreading from damaged trapping sites as a consequence of NanoSIMS analysis.

**Figure 5:**
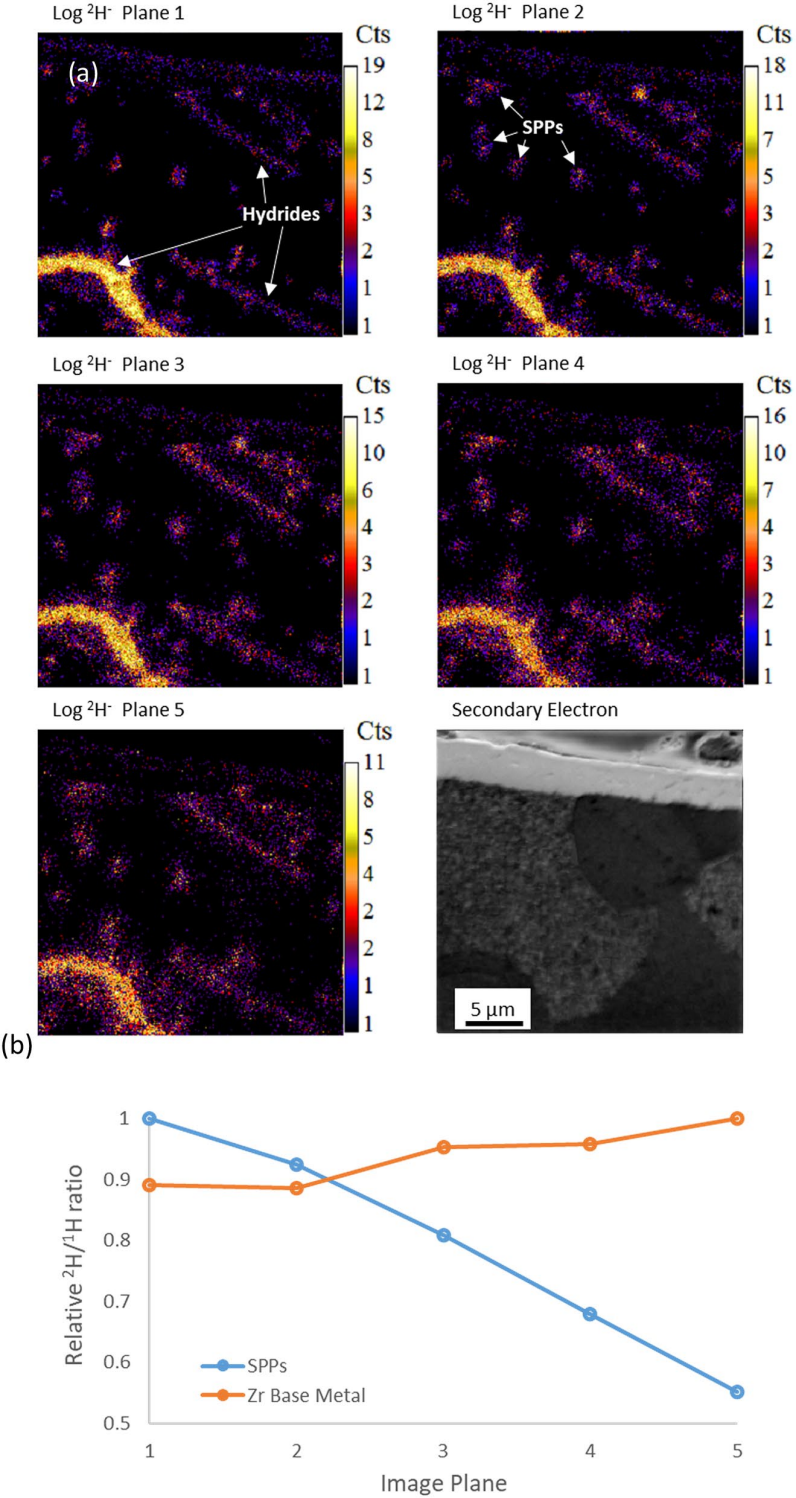
(**a**) NanoSIMS ^2^H images from the cross section of a Zry-2 sample corroded for 283 days in autoclave, 5 consecutive image planes and the SE image. The hydride and the enrichments around the SPPs become diffuse and the signal strength decreases with increasing image depth. (**b**) Shows the relationship between image plane and ^2^H/^1^H ratio around the SPPs and in the α-Zr base metal.

To demonstrate this effect, Fig. 5 shows five successive image acquisitions from a 5th ROI on a 283 day Zy-2 sample. In this ROI a widespread pattern of ^2^H concentrations is visible around SPPs, consistent with the observed ^2^H distributions seen in Figs. 1 and 2. However in this ROI there are also a number of hydrides present, these are indicated in the image, and are easily identifiable due to the high level of ^2^H present and their elongated morphology which is different to the concentrations of ^2^H around SPPs. With successive planes of analysis, the ^2^H signals around SPPs reduce in intensity and become diffuse. A similar effect can be seen for the three hydrides as they are also damaged by the Cs^+^ beam. The graph in Fig. 5 was produced by generating ROIs around each ^2^H concentration using the method described previously with L’image, however it should be noted that any ROIs that intersected with the oxide layer or the hydrides present were discarded. The data plotted in Fig. 5 were generated by summing the total ^1^H^−^ and ^2^H^−^ counts across all the ROIs in each plane and then finding the isotopic ratio. The graph shows a relative decrease in the ^2^H/^1^H ratio of the SPPs between image planes 1 and 5, showing that ^2^H is released from its trapping sites by Cs^+^ ion bombardment. The small relative increase in the ^2^H/^1^H ratio for the surrounding Zy-4 base metal indicates that the ^2^H is not only released to the vacuum but also migrates laterally onto the Zy-4 surface.

**Figure 6:**
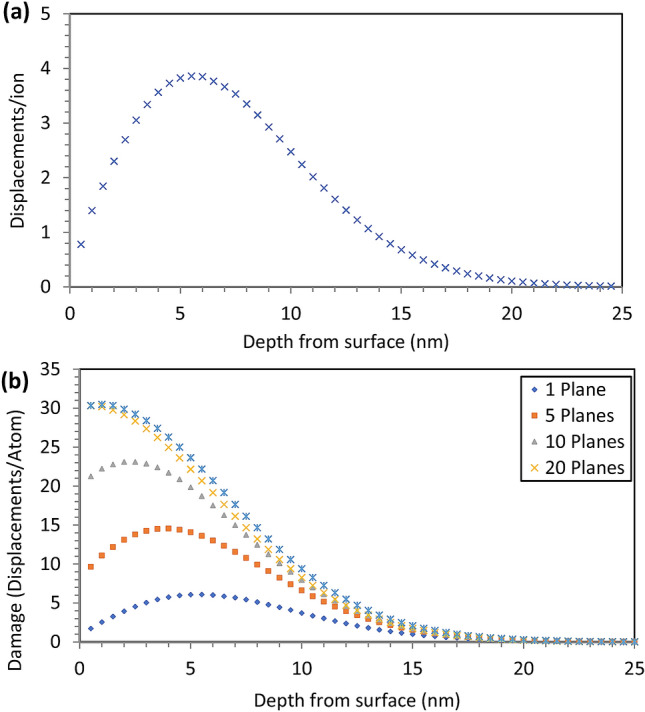
(**a**) Graph showing the irradiation damage distribution with depth after a 16 keV Cs atom strikes a Zry-4 target, as modelled by SRIM. (**b**) Shows the accumulation of damage in a Zy-4 target for increasing numbers of NanoSIMS analysis planes assuming a current of 1.5 pA, dwell time of 0.005 s, 256 × 256 pixels over a 30 μm raster size and a 1 nm depth removal rate per plane.

This observed depletion and dispersion of the ^2^H^−^ signal is due to the accumulation of sub-surface damage in the sample, which is a result of continued analysis with the 16 keV Cs^+^ primary ion beam. Figure 6a plots displacements produced by a single 16 keV Cs^+^ ion impacting a Zy-4 target with depth, calculated using quick Kinchin-Pease calculations in SRIM^59^. The damage caused by each ion impact extends far beneath the ~ 1 nm sampling depth produced by each impact^60^. As a result, damage accumulates in the sample with successive planes of analysis. Figure 6b shows how the damage rapidly accumulates with continued analysis, using similar analysis conditions to those used in this paper, before saturating at ~ 30 planes of analysis.

These results indicate that NanoSIMS images are always acquired from heavily damaged material. As it can take > 5 min to image a single plane, any hydrogen released from the damaged trap sites has time to redistribute to the surrounding α-Zr matrix before being analysed in a subsequent image plane. This is the main cause of the depletion/degradation of ^2^H^−^ signal observed in Fig. 6 and contributes significantly to the large measured size of the ^2^H features given in Table 1. Furthermore, this damage explains why the present result has not been observed in previous analysis undertaken with the NanoSIMS. If the imaging conditions are not correctly chosen, all ^2^H will have diffused away from the sample prior to imaging.

This effect is a particular concern for SIMS imaging of hydrogen in the α-Zr matrix as hydrogen is very mobile in the metal matrix, even at room temperature^11^. This effect is of less important when imaging hydrogen in the oxide (as in^34^) as the hydrogen is much less mobile and will not redistribute as readily.

## Conclusions

Using NanoSIMS and EDS, it has been observed that SPPs in Zy-2 and Zy-4 can act as preferential trapping sites for ^2^H introduced via corrosion, with measured isotope ratios of ^2^H/^1^H = 1.8–8.2 × 10^–2^, far in excess of the natural ratio. Complementary DFT modelling of the SPPs in Zy-2 and Zy-4 has shown that H solution in the bulk of the SPPs is thermodynamically unfavourable indicating that the observed ^2^H trapping occurs in the α-Zr matrix around the SPPs. This trapping is likely the result of tensile thermal mismatch stresses generated in the matrix around the SPPs by the differences in thermal expansion coefficients between the matrix and the SPP on cooling.

As current understanding of the hydrogen within Zr alloys does not take hydrogen segregation to SPPs into account, and factors such as the hydriding behaviour are crucial pieces of information from a safety perspective, this new observation can inform models used to predict the margins of safety in Zr alloy components for nuclear applications, thus ensuring safe and efficient operation.

## Methods and materials

### Experimental

Two different sets of zirconium alloys were analysed, one Zy-4 sample and two Zy-2 samples. The samples were oxidised in autoclaves at Jacobs, first in a refreshed autoclave at 350 °C and 18.6 MPa for 341.0 (Zy-4), 222.4 (Zy-2) and 3.3 (Zy-2) days, before being transferred to a static autoclave spiked with 50% ^2^H_2_O for 61.2 days (Zy-4 and Zy-2) and 15% ^2^H_2_O for 45.0 days (Zy-2) in otherwise similar conditions. These particular samples were chosen from an available array of pre-oxidised samples made available for this study, oxidation and weight gain data was provided by Jacobs plc.

The oxidation conditions were chosen due to their similarity to the conditions found in pressurised water reactors but with a slightly elevated temperature to increase the corrosion rate. Deuterium enriched water was used to allow unambiguous identification of hydrogen species introduced during the final oxidation step. The weight gains and estimated oxide thicknesses for the samples used in this study can be found in Table 2**.**

**Table 2:** Samples produced for this study showing the enrichment level of the water, oxidation time and oxide thickness.

Alloy	Dopant	Oxidation time (days)			Oxide thickness (μm)		
		Refreshed autoclave	Spiked autoclave	Total	Refreshed autoclave	Spiked autoclave	Gain
Zr-2	15% ^2^H_2_O	3.3	45.0	48.3	0.76	1.41	0.65
Zr-2	50% ^2^H_2_O	222.4	61.2	283.6	2.76	3.91	1.15
Zr-4	50% ^2^H_2_O	341.0	61.2	402.2	4.46	5.43	0.97

Each sample was sectioned into 2–3 mm cubes and mounted in cross section using araldite resin such that the full thickness across the oxide layer was visible. The samples were prepared by mechanical grinding from 800 to 4000 grit followed by a slow polishing process using a colloidal silica (0.04 μm) suspension for > 30 min, this preparation technique is based on the method used by Yardley et al.^33^. Immediately prior to loading into the NanoSIMS, the samples were coated with 10 nm of platinum in order to prevent charging of the samples during analysis.

The analysis was performed on a CAMECA NanoSIMS 50L (Cameca, France), which is a double focussing mass spectrometer with one fixed and six movable detectors and a separate secondary electron (SE) detector. A 16 keV Cs^+^ ion beam with a current of 1.3–1.8 pA was used to sputter the surface and generate negative secondary ions. All regions of interest were initially implanted with a high current beam to remove the Pt coating in the analysis area and increase secondary ion yield and then 5–10 sequential images were acquired from each region of interest. The settings for each individual acquisition are given in the relevant figure captions with the Energy Slit (ES) set to 3 (20 µm) and Aperture Slit (AS) set to 2 (200 µm) for each analysis. Image analysis was carried out using L’image (LR Nittler, Carnegie Institution of Washington).

SPPs in regions analysed using the NanoSIMS were identified using a Zeiss Merlin Field Emission Gun-Scanning Electron Microscope (FEG-SEM) operating at 3 kV for a high lateral resolution sufficient to detect the SPPs. SE images were obtained using the InLens detector. The SPPs were chemically identified using the Oxford Instruments Extreme detector which uses a windowless silicon-drift detector developed for low-kV energy dispersive X-ray spectroscopy (EDS) analysis.

### Modelling

The solution enthalpy of H in Zr and its intermetallics, from H2 molecules, is negative^25^ (i.e. hydriding is a exothermic). Thus, we measure the relative enthalpy ($\Delta{\text{ H}}_{sol}$) between H solution in the SPPs and H solution in α-Zr matrix:

$$\Delta{\text{ H}}_{sol}={(E}_{\alpha -Zr}^{DFT}+{E}_{SPP+H}^{DFT})-\left({E}_{\alpha -Zr+H}^{DFT}+{E}_{SPP}^{DFT}\right),$$

where ${E}_{\alpha -Zr}^{DFT}$ and ${E}_{SPP}^{DFT}$ are the DFT energies of hcp-Zr and SPP supercells (as described below), and ${E}_{\alpha -Zr+H}^{DFT}$ and ${E}_{SPP+H}^{DFT}$ are the DFT energies of the same structures with the addition of one H interstitial defect (for hcp-Zr we considered only the more favourable tetrahedral site).

The ternary Zr(Fe,Cr)2 SPPs found in Zy-2 and Zy-4 are C-14 Laves phases with hexagonal structure (space group P63/mmc) and disorder only on the Fe/Cr sublattice. These were modelled using a set of special quasi-random structures (SQS)^61^. SQS reproduce, in a finite supercell, the radial correlation functions of an infinite and perfectly disordered crystal. SQS were generated with compositions as close as possible to the experimental reports, giving Fe/(Fe + Cr) ratios of 43.72% and 62.5% respectively.

12 SQS were generated for each composition using the MCSQS script from the ATAT toolkit^62^. The objective function included all pair-wise correlations up to 4.343 Å (five clusters) and triplet correlations up 2.767 Å (two clusters). Supercells containing 96 atoms were used to ensure a good representation of disorder, representative compositions, and small finite size effects.

While it is known that H preferentially occupies tetrahedral sites in Zr^41,63^ (both hcp and bcc) and in (ordered) binary Zr intermetallics^25^, it is unclear whether this would be the case in disordered ternary SPPs. Thus 35 different interstitial sites were randomly selected, for each SQS composition, to probe a representative sample of interstitial environments with no bias.

DFT calculations were performed with the VASP package^64^ using a planewave basis set, combined with the PBE exchange–correlation functional^65^ pseudopotentials containing 1s1 and 4s24p65s24d2 valence electrons for H and Zr respectively, and a consistent energy cut-off of 350 eV. SQS cells were relaxed at constant pressure until the energy difference was below 10–5 eV. H defects were relaxed in the lowest energy SQS at constant volume with the same energy convergence criteria. K-point sampling was performed with a 4 × 4 × 2 Γ-centred grid with Methfessel–Paxton band spearing of 0.01 eV. All simulations were spin-polarised.
